# Effects of Material Structure on Stress Relaxation Characteristics of Rapidly Solidified Al-Fe Alloy

**DOI:** 10.3390/ma16175949

**Published:** 2023-08-30

**Authors:** Ryohei Kobayashi, Tatsuya Funazuka, Toru Maeda, Tomomi Shiratori

**Affiliations:** 1 Advanced Materials Laboratory, Sumitomo Electric Industries, Ltd., Itami-shi 664-0016, Japan; 2 Graduate School of Science and Engineering, University of Toyama, Toyama-shi 930-8555, Japan; 3 Academic Assembly Faculty of Engineering, University of Toyama, Toyama-shi 930-8555, Japan

**Keywords:** aluminum alloy, electrical conductor, powder metallurgy, stress relaxation, mechanical property

## Abstract

An Al-Fe alloy which was produced by hot extrusion of rapidly solidified powder is a possible solution to substitute copper-based electrical conductor material due to its high strength and high electrical conductivity. However, the stress relaxation characteristic—an essential parameter as a conductor material—and the effect of the material structure have not been reported, which was the aim of the present paper. An Al-5%Fe alloy was selected as the test material. The material structures were controlled by hot extrusion practice, annealing, and cold rolling. The Al-Fe intermetallic compound particles controlled the residual stress after the stress relaxation test via the Orowan mechanism. Decreasing the mean inter-particle distance reduces the electrical conductivity. The increase in the number of dislocations by the cold rolling increased strength at room temperature without changing electrical conductivity; however, it did not have a positive effect on the stress relaxation characteristics. The stress relaxation characteristics and the electrical conductivity of the Al-Fe alloy were superior to conventional C52100 H04 phosphor bronze when compared with the case of the same mass.

## 1. Introduction

Recently, since smartphones have become larger and the types of wearable devices have increased in variety, their weight reduction has become important. The electrical conductivity and strength per unit mass of aluminum is twice as high as that of copper, and so, using aluminum as a conductive material contributes to weight reduction [1]. Another reason for replacing copper with aluminum is the uncertain future of copper reserves [2]. However, aluminum tends to undergo stress relaxation, i.e., creep deformation, at high temperatures such as those used in connectors for high-current circuits, and so, aluminum materials with high strength are required [3,4]. Currently, C5210 H04 phosphor bronze, which has excellent spring properties, is used for the female terminals of mobile phone connectors [5]. Therefore, an aluminum alloy having a stress relaxation resistance comparable to that of phosphor bronze is desired.

Since copper is often used as a conductive material, there are many examples of research papers on its stress relaxation properties [6,7]. However, there have been few reports on the stress relaxation properties of aluminum. Recently, rapidly solidified Al-Fe alloys have been investigated as materials to replace copper-based conductive materials [8,9,10]. It has been reported that the Al-Fe alloys exhibit excellent creep properties at high temperatures above 700 K [11], but there are no reports on the stress relaxation properties of the alloys around 393–423 K, where conductive materials are used. It is important to know the stress relaxation properties of such an alloy and the effect of material structure in order to use it as a conductive material. Regarding the strength of rapidly solidified Al-Fe alloys at room temperature, finely dispersed Al-Fe intermetallic compound particles contribute to particle dispersion strengthening [9]. The Al-Fe dispersoids transforms from a metastable fine Al_6_Fe phase to a coarse Al_13_Fe_4_ phase (also called Al_3_Fe) when heated above 623–673 K [12]. This temperature range is close to the temperature of the hot extrusion, which is one of the major methods of producing rapidly solidified Al-Fe alloys [8,10]. Hence, the extrusion temperature may affect the strength of a rapidly solidified Al-Fe alloy. However, the effects and mechanisms are unknown. Furthermore, methods of processing such as cold rolling and drawing and annealing are considered to adjust the strength of Al-Fe alloys. It is of industrial importance to know their influence.

In the present study, we investigated the effects and mechanisms of the material structure on the stress relaxation characteristics of a rapidly solidified Al–Fe alloy by changing the hot extrusion temperature, annealing temperature, and cold rolling conditions.

## 2. Materials and Methods

**Figure 1:**
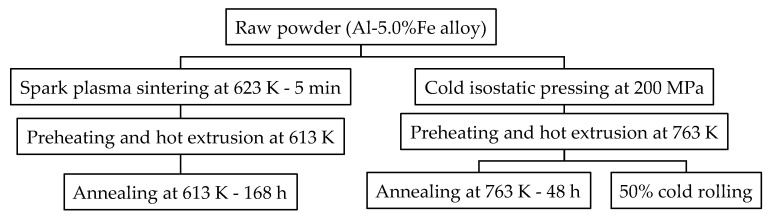
Experimental procedure for sample preparation.

In order to change the distribution of the Al-Fe intermetallic compound, the extrusion conditions of the Al-Fe alloy material were varied. The sample preparation method is shown in Figure 1. An air-jet atomized Al-5.0%Fe alloy powder manufactured by Toyo Aluminium K.K. (Osaka, Japan) was used as the raw material. The impurities in the alloy are 0.1% or below. The average particle size of the powder is approximately 30 μm. Samples aiming at fine material structure were produced by spark plasma sintering and hot extrusion. The powder was spark plasma, sintered into a cylindrical body 42 mm in diameter and 23 mm in height under the conditions of a temperature of 623 K and a pressurization of 20 MPa. Three pieces of this sintered body were preheated and then hot-extruded at 613 K into a round bar with a diameter of 8 mm. Samples aiming at coarse material structure were produced by cold compacting and hot extrusion at relatively high temperatures. The powder was compacted into a cylindrical shape with a diameter of 170 mm and a height of 300 mm by cold isostatic pressing with a pressure of 200 MPa. The billet was preheated and then hot-extruded at 763 K into a plate with a thickness of 5 mm and a width of 30 mm. The relative density of the extrudates (relative to the density of casts) measured by Archimedes’ method was 0.99 or more in all extrusion conditions, which confirmed that the extrudates were completely densified. Some of the Al-Fe alloys were annealed at 613 K–168 h or 763 K–48 h. It is expected that the lower the heat treatment temperature is, the slower the structural change will be. Therefore, the heat treatment time at 613 K was longer than that at 763 K. In order to understand the effect of cold working, a plate sample extruded at 763 K was subjected to multi-pass cold rolling with a total reduction of 50%. As comparative materials, data for C10200 H04 oxygen-free copper [13,14], C26000 H04 brass [13,15], C52100 H04 phosphor bronze [13,16], 1050-H24 aluminum [1], and 6101-T6 aluminum alloy [1] were used.

The 0.2% proof stress was measured by using a tensile test. A specimen with a thickness of 1 mm, a width of 8 mm, and a length of 100 mm was ground from the Al-Fe alloy so that the longitudinal direction coincided with the extrusion direction. The electrical conductivity was measured by using a vortex conductometer Sigmatest 2.069 (Foerster Japan Limited, Tokyo, Japan). The microstructures were observed by FE-SEM (JEOL JSM-7800F, Tokyo, Japan) at an accelerating voltage of 5 kV and WD = 10 mm on the cross-sections parallel to the extrusion direction of the extrudate. The images were binarized and analyzed by ImageJ 1.53 k, which is an open-source image processing software, to find the area fraction $A_{f}$ (equivalent to volume fraction $V_{f}$) and the average radius of the particles r. The radius of the particles was obtained as half the equivalent circle diameter of them. In this analysis, particles below an equivalent circle diameter of 0.1 μm (equivalent to 5 pixels or below) were excluded as noise. To understand the grain structure, the cross-sections parallel to the extrusion direction of the extrudate were observed and analyzed by FE-SEM (ZEISS Gemini 450, Jena, Germany) and EBSD (Oxford Symmetry, Oxford Instruments, Abingdon, UK). In the analysis, the interfaces with an inclination of 5° or more were considered grain boundaries and the area-weighted average of the grain size was obtained.

**Figure 2:**
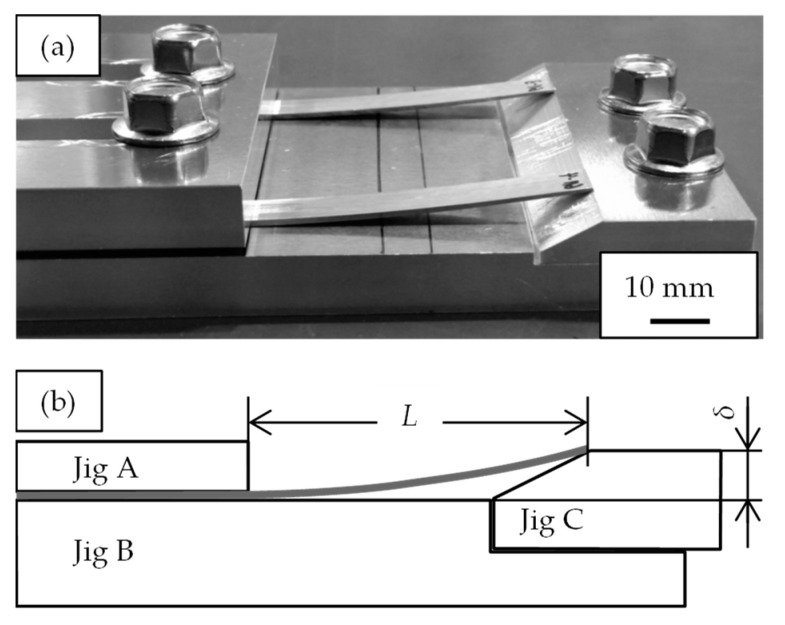
Measurement method of stress relaxation property: (**a**) schematic diagram and (**b**) picture of the jigs and the samples.

The test conditions for evaluating stress relaxation characteristics are as follows, according to the technical standard [17]. A specimen with a thickness of 0.6 mm, a width of 8 mm, and a length of 100 mm was ground from the Al-Fe alloy so that the longitudinal direction coincided with the extrusion direction. The samples were attached to the jig as shown in Figure 2. From the cantilever beam formula, the maximum bending stress σ of this sample is expressed by the following equation [17]:

(1) $$\sigma =\frac{1.5Eth}{L^{2}}$$

where *E* is the modulus of elasticity, *t* is the thickness of the sample, *h* is the initial deflection height, and *L* is the distance from the edge of jig A to the edge of the sample. The modulus of elasticity *E* was measured according to the technical standards [18]. The height *h* was fixed at 5 mm. The length *L* was adjusted so that the maximum bending stress *σ* was 80% of the 0.2% proof stress. The samples were exposed at 393 K or 423 K for 24–1000 h and then brought to room temperature. The deflection height $h_{t}$ remaining in the sample after removing the jig C was measured. This measurement was repeated using the same sample and jig. The stress relaxation rate $r_{sr}$ is obtained by using the following equation [17]:

(2) $$r_{sr}\left(\%\right)=\frac{h_{t}}{h}\times 100$$

In order to use the aluminum alloys for the connection part of male–female terminals or bolted terminals, it is necessary to maintain high contact pressure between the terminals [4]. As the index of the contact pressure, the residual stress $\sigma_{r}$ remaining after the stress relaxation test was used.

(3) $$\sigma_{r}=\sigma_{y}\left(1-r_{sr}\right)$$

Here, $\sigma_{y}$ is the initial 0.2% proof stress before the stress relaxation test. An increase in this value is considered to lead to an improvement in the contact pressure.

## 3. Results

**Figure 3:**
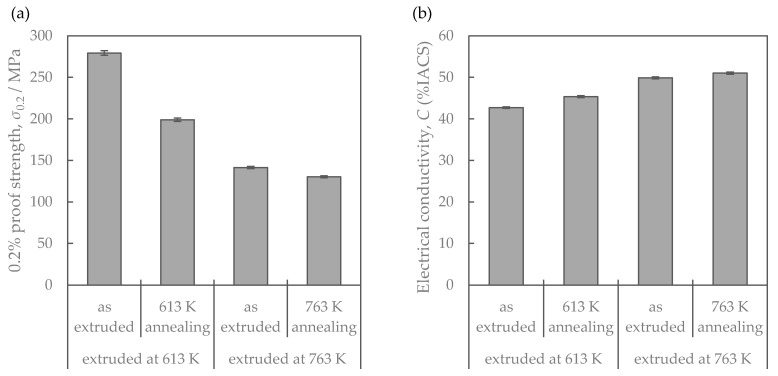
Properties of the Al-Fe alloys: (**a**) mechanical property and (**b**) electrical conductivity.

**Figure 4:**
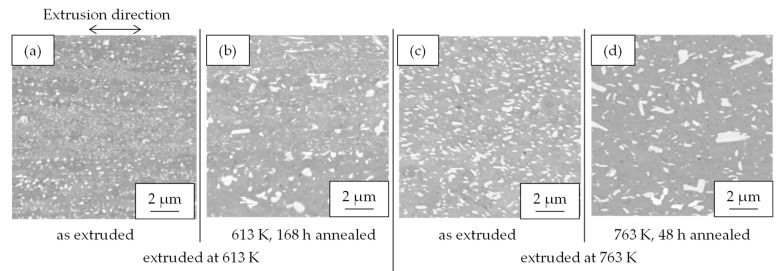
Cross-sectional SEM-COMPO images of Al-Fe alloys (**a**) extruded at 613 K, (**b**) extruded at 613 and then 613 K—168 h annealed, (**c**) extruded at 763 K, and (**d**) extruded at 763 K and then 763 K—48 h annealed.

**Figure 5:**
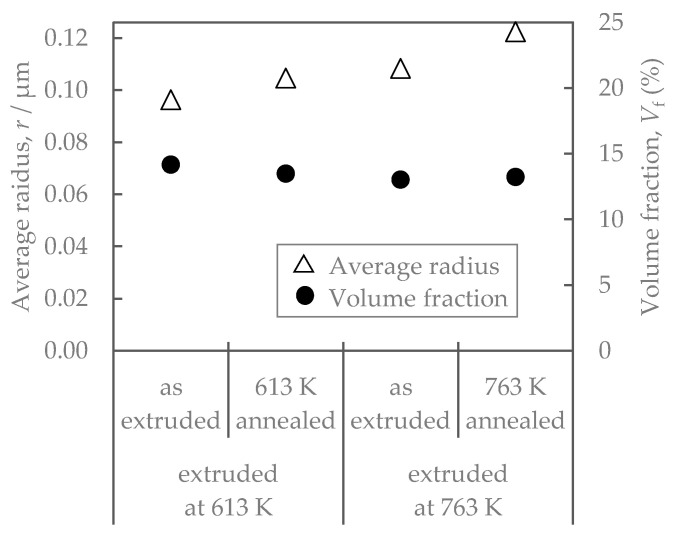
Volume fraction and average radius of second-phase particles in Al-Fe alloys before and after annealing.

**Figure 6:**
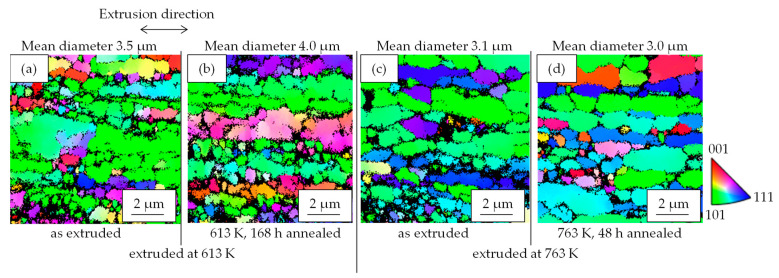
Cross-sectional grain maps obtained by SEM-EBSD of Al-Fe alloys (**a**) extruded at 613 K, (**b**) extruded at 613 and then 613 K—168 h annealed, (**c**) extruded at 763 K, and (**d**) extruded at 763 K and then 763 K—48 h annealed.

Figure 3 shows the properties of Al-Fe alloys with different extrusion and annealing temperatures. The higher the extrusion temperature is, the lower the strength and the higher the electrical conductivity are. Additional heat treatment had similar effects, resulting in a decrease in strength and an increase in conductivity. Figure 4 shows their SEM-compo images. The distributions of the Al-Fe intermetallic compound particles, which appear white in the images, differed depending on the differences in the extrusion temperature and the annealing temperature, which was intended. Figure 5 shows the volume fraction $V_{f}$ and average particle radius r of the second-phase particles obtained by analyzing Figure 4. The particles were the smallest when the extrusion temperature was as low as 613 K, and became coarser at higher extrusion temperatures and with the annealing. There was no significant difference in the volume fraction of the particles. Figure 6 shows their EBSD-IPF images. All samples had coarse grains of 2 μm or more and fine grains of 1 μm or less. The area-weighted average grain size was the same: 3–4 μm.

**Figure 7:**
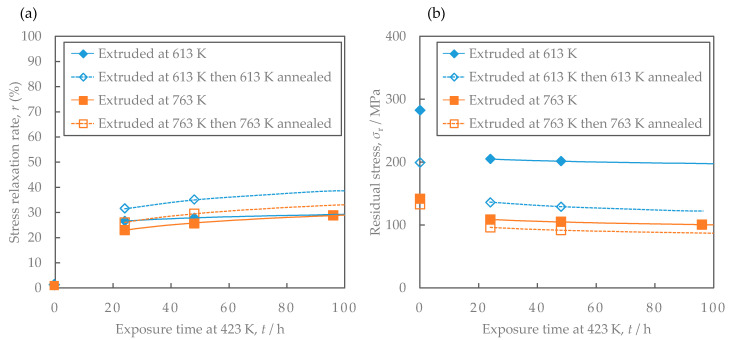
Comparison of the stress relaxation properties of Al-Fe alloys at 423 K before and after annealing: (**a**) stress relaxation rate and (**b**) residual stress. The lines are regression curves proportional to the logarithm of the exposure time.

Figure 7 shows the stress relaxation properties. The stress relaxation rate increased slightly with time, and the stress relaxation rate was about 30–40% for up to 100 h. The lower the extrusion temperature was, the higher the stress relaxation rate was. Annealing increased the stress relaxation rate. Figure 7b shows the residual stress after the stress relaxation test considering the initial 0.2% proof stress. The lower the extrusion temperature was, the higher the residual stress was. Annealing reduced the residual stress.

**Figure 8:**
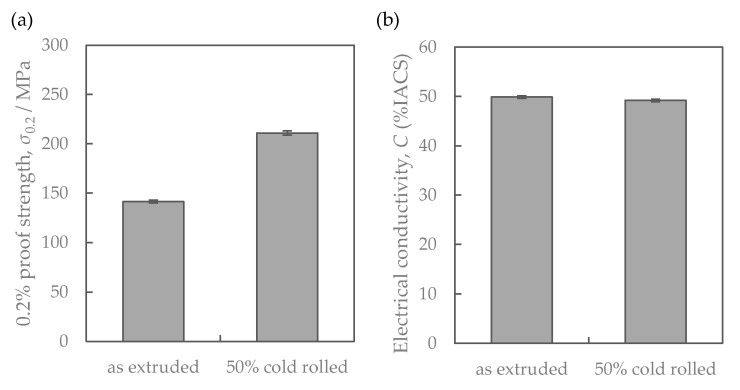
Properties of the Al-Fe alloys extruded at 763 K before and after cold rolling: (**a**) mechanical property and (**b**) electrical conductivity.

**Figure 9:**
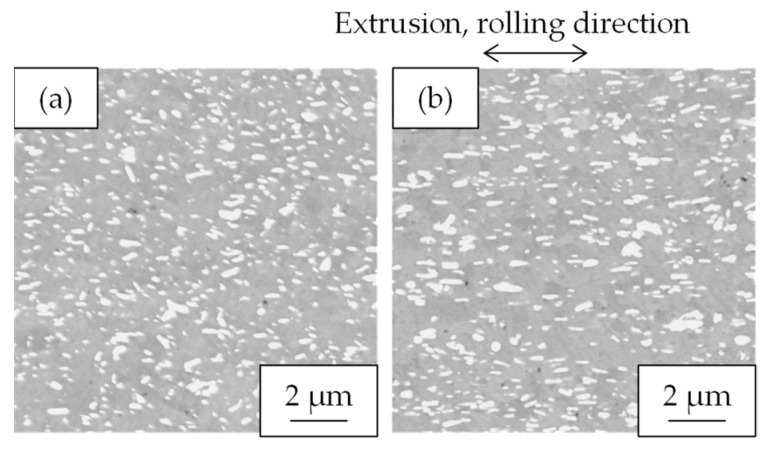
Cross-sectional SEM-COMPO images of Al-Fe alloys (**a**) extruded at 763 K and (**b**) extruded at 763 K and then 50% cold-rolled.

**Figure 10:**
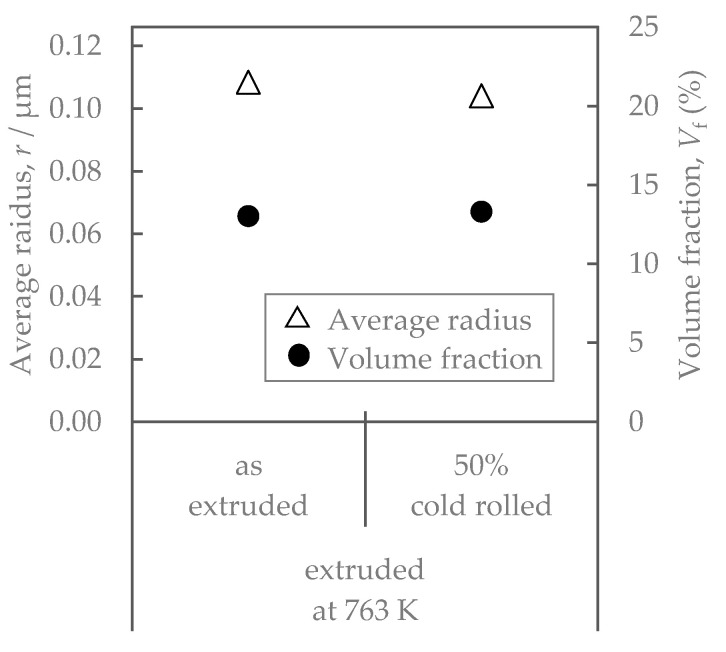
Volume fraction and average radius of second-phase particles in Al-Fe alloys before and after cold rolling.

**Figure 11:**
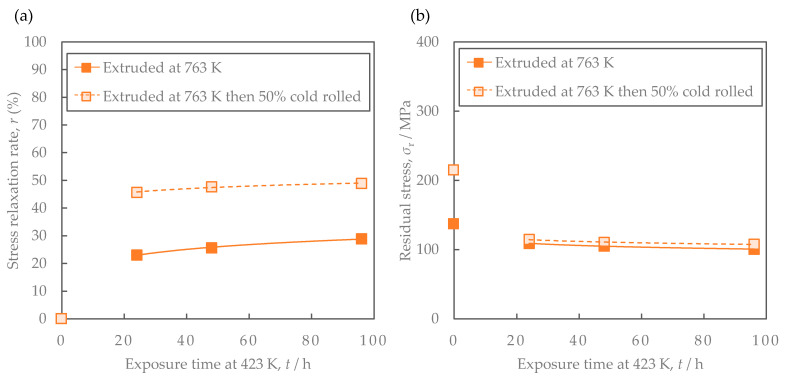
Comparison of the stress relaxation properties of Al-Fe alloys at 423 K before and after cold rolling: (**a**) stress relaxation rate and (**b**) residual stress. The lines are regression curves proportional to the logarithm of the exposure time.

Figure 8 shows the properties of the Al-Fe alloy extruded at 763 K and cold-rolled at a total rolling reduction of 50%. Cold rolling increased the strength while the electrical conductivity remained almost unchanged. Their SEM-compo images are shown in Figure 9, and their image analysis results are shown in Figure 10. The size of the second-phase particles of the cold-rolled material was slightly finer than that of the as-extruded material, and the volume fractions were almost the same. Figure 11 shows their stress relaxation properties. The stress relaxation rate of the cold-rolled material was higher than that of the as-extruded material. As a result, there was almost no difference between their residual stress levels.

**Figure 12:**
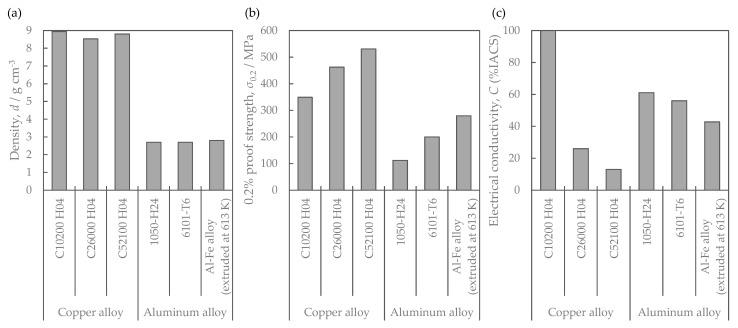
Properties of copper and aluminum materials: (**a**) density, (**b**) mechanical property and (**c**) electrical conductivity. Properties except for the Al-Fe alloy have been cited from the references [1,13,14,15,16].

**Figure 13:**
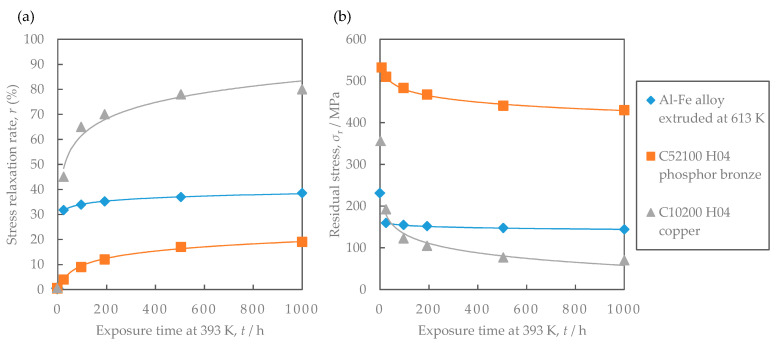
Comparison of the stress relaxation properties at 393 K: (**a**) stress relaxation rate and (**b**) residual stress. Properties of C26000 H04 brass and C52100 H04 phosphor bronze have been cited from reference [13]. The lines are regression curves proportional to the logarithm of exposure time.

Figure 12 shows the density, strength, and electrical conductivity of the Al-Fe alloy extruded at 613 K, which had the highest strength so far, and the comparative copper and aluminum materials. The strength of the Al-Fe alloy was lower than that of copper and higher than that of other aluminum alloys. Its electrical conductivity was higher than that of brass and phosphor bronze. Figure 13 shows the stress relaxation properties of the Al-Fe alloy, C10200 H04 oxygen-free copper, and C5210 H04 phosphor bronze. The oxygen-free copper is often used for bolted bus bars and the phosphor bronze is used for connector terminals. The exposure temperature for this measurement was 393 K and the time was 1000 h to simulate a practical environment when used as connector terminals. When the exposure time was more than 100 h, the residual stress of the Al-Fe alloy was higher than that of oxygen-free copper. On the other hand, the residual stress of the Al-Fe alloy was smaller than that of the phosphor bronze.

## 4. Discussion

### 4.1. Effect of Changes in Particle Distribution by Increasing Extrusion Temperature and Annealing

**Figure 14:**
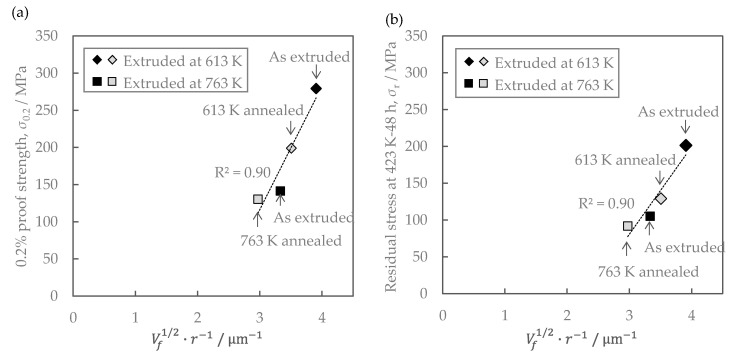
Relationship between distribution of secondary-phase particles and mechanical properties of Al-Fe alloy: (**a**) 0.2% proof strength of as-extruded materials; (**b**) residual stress after the exposure at 423 K for 48 h.

The residual stress after the stress relaxation test of the Al-Fe alloy changed with the increasing extrusion temperature and annealing (Figure 7). We consider the effect of the material structure. As shown in Figure 6, there is no significant difference in the grain size of the aluminum matrix, suggesting that its effect on residual stress is small. As shown in Figure 5, the volume fraction of the second-phase particles was almost the same in all samples, suggesting that the solid solution and dispersed amounts of iron were almost in an equilibrium state and that their effect on the residual stress was small. From Figure 5, the average radius of Al-Fe intermetallic compound particles varies with extrusion temperature and annealing, which may be related to residual stress. Assuming the Orowan strengthening mechanism by fine dispersoids, the strengthening contribution by fine Al-Fe dispersoids ∆σ would be roughly evaluated thus [19]:

(4) $$∆\sigma \propto V_{f}^{1/2}r^{-1}$$

where $V_{f}$ is the volume fraction and r is the average radius of the Al-Fe dispersoids. Figure 14 shows the relationship between the mechanical properties and the contribution of particle dispersion strengthening, where the right sides of Equation (4) are employed as an index. Both the as-extruded 0.2% proof stress and the residual stress after the stress relaxation test showed a linear relationship with $V_{f}^{1/2}r^{-1}$, which was found to follow the Orowan strengthening mechanism. Therefore, it is suggested that dispersoids inhibit dislocation motion not only at room temperature but also at high temperature and improve residual stress. The reason why the residual stress decreased after the stress relaxation test is that the dispersed particles became coarser due to the increase in extrusion temperature and annealing, and the ability of these particles to inhibit dislocation motion was weakened. Furthermore, these results provide an important indication that dislocation motion is involved in the stress relaxation process in the Al-Fe alloys, i.e., dislocation creep is dominant in the process. That is why increasing the dislocation pinning force by using other methods, such as refining the dispersoids by using severe plastic deformation [20], may further increase the residual stresses. These finding are useful for the further improvement of the design of the Al-Fe alloys.

**Figure 15:**
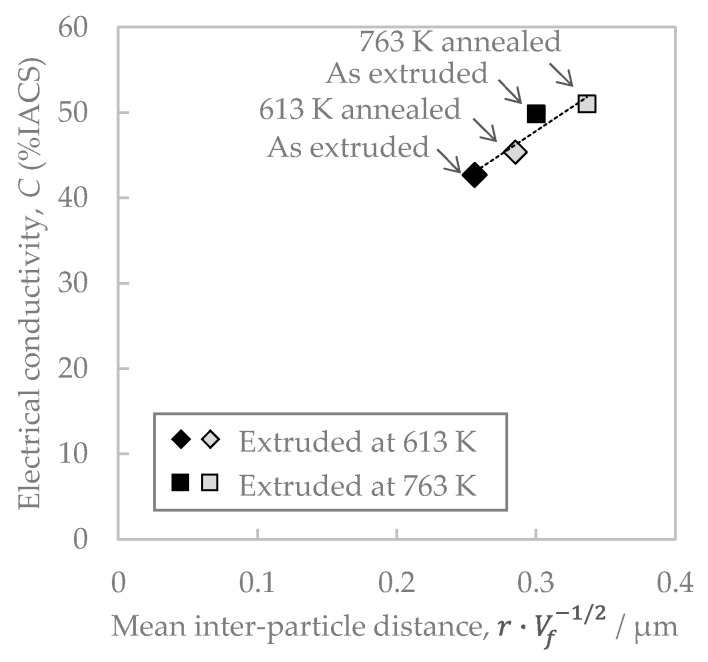
Relationship between electrical conductivity and mean inter-particle distance in Al-Fe alloy.

Next, we consider the effect of the distribution of the second-phase particles in the Al-Fe alloy on the electrical conductivity. Defects in aluminum, such as impurities and grain boundaries, scatter free electrons, reducing the electrical conductivity [21]. Al-Fe dispersoids can cause this free electron scattering. Thus, the mean particle spacing is estimated as being $r\cdot V_{f}^{-1/2}$ [22], and the relationship with conductivity is shown in Figure 15. These relationships were linear, i.e., the conductivity decreased as the mean particle spacing decreased. This suggests that the increase in the number of the Al-Fe dispersoids might increase the defects, such as lattice strain and boundaries around them, promote the scattering of free electrons. Another factor that would lowers the conductivity is an increase in the amount of solid solute iron. However, as shown in Figure 5, there was no significant difference between the volume fractions of Al-Fe dispersed particles in the samples. Therefore, the difference in the amounts of solute iron due to the increasing extrusion temperature and annealing would be small. Therefore, it is considered that the influence of the change in the amount of solute iron on electrical conductivity is smaller than that of the Al-Fe dispersoids.

In conclusion, it was clarified that the residual stresses after the stress relaxation test and electrical conductivity are governed by the distribution of the Al-Fe dispersoids. It was found that the increasing extrusion temperature and annealing affect the distribution of the particles, decreasing the residual stress and increasing the electrical conductivity.

### 4.2. Effect of Cold Rolling

**Figure 16:**
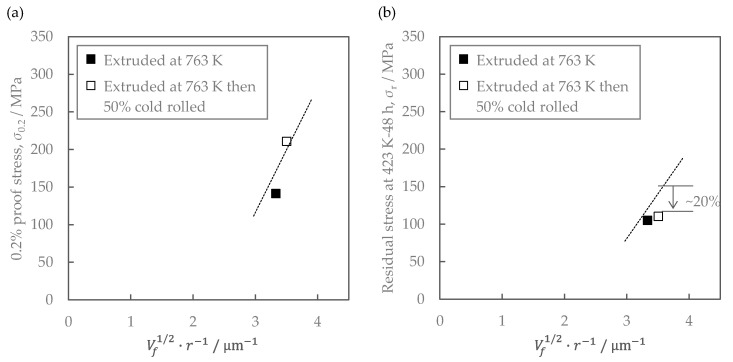
Relationship between distribution of secondary-phase particles and mechanical properties of Al-Fe alloy: (**a**) 0.2% proof strength of as-extruded materials; (**b**) residual strength after the exposure at 423 K for 48 h. The lines are the same as the regression lines in Figure 14.

Cold rolling increased the as-extruded strength without changing conductivity (Figure 8), but failed to improve the residual stress after the stress relaxation test (Figure 11). The effect of material structure was considered as follows. Figure 16 shows the relationship between the mechanical properties and the contribution of particle dispersion strengthening, where the right side of Equation (4) is employed as an index. The dotted lines in the figures are the same regression line as in Figure 14. The residual stress of cold-rolled material was about 20% lower than the regression line. This result clarified that the residual stress in the cold-rolled material cannot be explained only by the distribution of Al-Fe dispersoids as described in Section 4.1.

**Figure 17:**
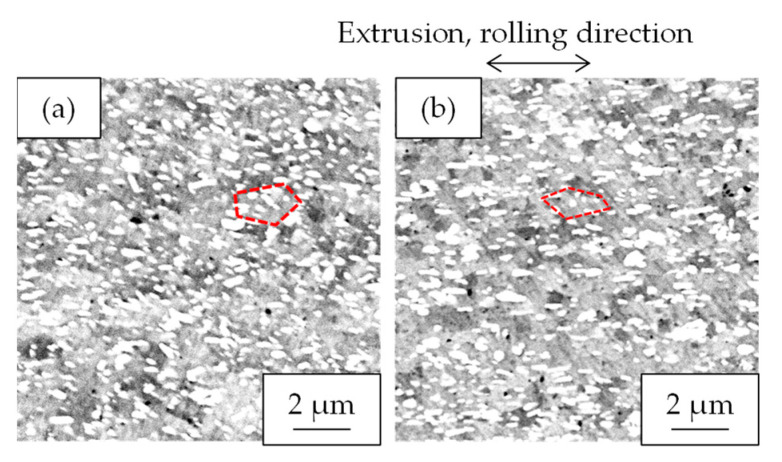
Cross-sectional SEM-COMPO images of Al-Fe alloy extruded at 763 K: (**a**) as-extruded and (**b**) additionally 50% cold-rolled. Dotted lines indicate examples of the typical grain boundary.

As factors for the decrease in residual stress after the stress relaxation tests in the cold-rolled material, the effects of grain size and dislocation density were considered. Figure 17 shows the cross-sectional SEM-compo images of the samples before and after rolling with the brightness and contrast adjusted to compare the grain sizes. Roughly estimating the grain sizes from the electron channeling contrast of the aluminum matrix, both were thought to be around 2–3 μm, and the change due to cold rolling seemed to be small. On the other hand, as shown in Figure 8, the 0.2% yield strength of the Al–Fe alloy after cold rolling was higher than that before rolling. This result suggests that the dislocation density was increased by the cold rolling. Dislocations induced by plastic strain can increase the creep deformation rate [23]. Since the stress relaxation is a type of creep deformation [24], the dislocations induced by the cold rolling in the Al-Fe alloy will also be associated with the stress relaxation. Additionally, the introduction of the dislocations by cold rolling will increase the residual stress in the Al-Fe alloy before the stress relaxation test. When exposed to the high temperature during the stress relaxation test, the dislocations introduced by the cold rolling will rearrange and be annihilated in order to reduce the strain energy, resulting in strain relaxation. The rearrangement and the annihilation of the dislocations might reduce the residual stress after the stress relaxation test. This will be a phenomenon similar to the relief of the residual stress after plastic deformation, as follows: post-deformation annealing enables residual stress relief via a modification of the dislocation substructure [25]. Mobile dislocations which would be introduced by the cold rolling could promote the stress relaxation as well [26]. Therefore, as found in Figure 11, the higher stress relaxation rate of the cold-rolled Al–Fe alloy than the as-extruded one is considered to be associated with such an introduction of the dislocations.

In conclusion, strengthening via the introduction of dislocations in cold rolling cannot improve the residual stress after the stress relaxation test, which is an industrially important finding.

### 4.3. Comparison of Stress Relaxation Characteristics of the Al-Fe Alloy with the Conventional Materials

As shown in Figure 13, the residual stress of the Al-Fe alloy after the stress relaxation test, for more than 100 h, was higher than that of oxygen-free copper. Oxygen-free copper is often used in bolted bus bars. Therefore, the Al-Fe alloys may be used as bus bars with excellent long-term connection reliability. The residual stress of the Al-Fe alloy after the stress relaxation test was smaller than that of the conventional phosphor bronze (Figure 13). Therefore, replacing phosphor bronze with the Al-Fe alloy as a conductor material requires increasing the cross-sectional area to maintain the same contact force. Since the Al-Fe alloys had a lower density than the phosphor bronze (Figure 12), the weight may be reduced even if the cross-sectional area was increased. In order to consider the effect of density, their specific strength s was compared as an index of their mechanical properties.

(5) $$s=\frac{\sigma }{\rho_{d}}$$

**Figure 18:**
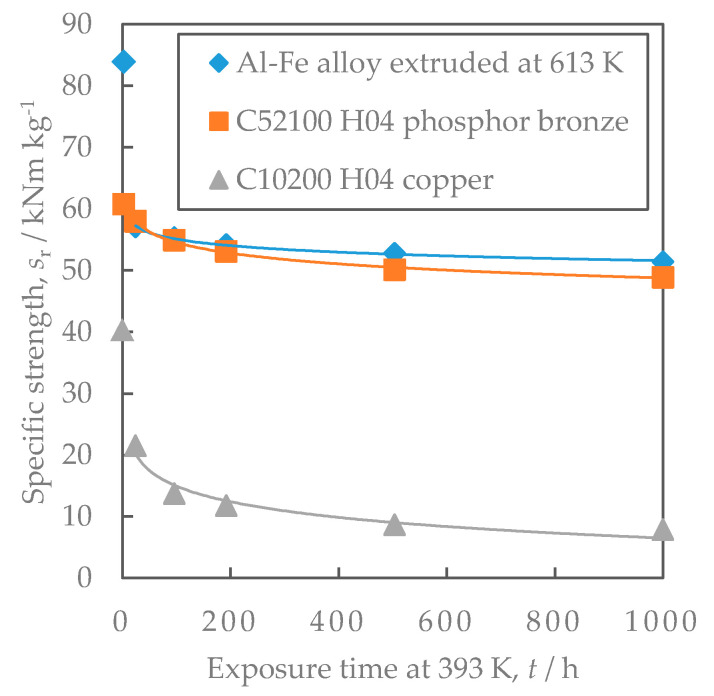
Comparison of the specific strength at 393 K. The lines are regression curves proportional to the logarithm of the exposure time.

Here, σ is the strength (0.2% proof stress or residual stress after the stress relaxation test) and $\rho_{d}$ is the density. The higher the specific strength s is, the higher the residual stress when the cross-sectional area is increased with the same mass will be. As shown in Figure 18, the specific strength of the Al-Fe alloy was slightly higher than that of C52100 phosphor bronze. Therefore, the residual stress of the Al-Fe alloys was found to be superior to the conventional phosphor bronze when the cross-sectional area was increased. Moreover, the conductivity of the Al-Fe alloy is more than twice higher than that of phosphor bronze (Figure 12c). Hence, the temperature rise due to the Joule heating when an electric current is applied to Al-Fe alloy is considered to be much lower than that of C52100 phosphor bronze, which makes it easier to pass more current. The lower the stress relaxation test temperature is, the higher the residual stress is [17]. Considering these factors, the stress relaxation of the Al-Fe alloy would be smaller than that of phosphor bronze when the current is applied to the same cross-sectional area. Thus, the required cross-sectional area of the Al-Fe alloy can be reduced, which is expected to contribute to weight reduction in the conductive material. Materials other than phosphor bronze for connector terminals include Cu-Ni-Si alloys and Cu-Be alloys [6]. Their stress relaxation properties are superior to those of phosphor bronze, allowing for use in higher-temperature environments of 150 °C and above [5]. In order to use the Al-Fe alloy in such an environment, it is considered effective to reduce the extrusion temperature of the Al-Fe alloy for the further refinement of the Al-Fe dispersoids, which should be validated in a future study.

## 5. Conclusions

We investigated the effects of material structure on the stress relaxation properties and the electrical conductivity of rapidly solidified Al-Fe alloys and obtained the following findings.

1. The distribution of Al-Fe intermetallic compound particles affects the conductivity and residual stress after the stress relaxation tests. The particles control the residual stress via the Orowan mechanism. Decreasing the mean inter-particle distance reduces the conductivity. The increasing in extrusion temperature and the annealing affect the particles’ distribution, with higher extrusion temperatures and more annealing reducing the residual stress and increasing the conductivity.
2. The cold rolling of the Al-Fe alloys can increase strength at room temperature without changing electrical conductivity. However, in the study, the cold rolling did not have a positive effect on the stress relaxation characteristics of the Al-Fe alloy. The dislocations induced by the cold rolling would be associated with the effect on the stress relaxation characteristics.
3. The residual stress after the stress relaxation test of the Al-Fe alloy was lower than that of C52100 H04 phosphor bronze. However, considering its density, the Al-Fe alloy is superior when compared with the case of the same mass. Since the conductivity of the Al-Fe alloy is more than twice as high as that of the phosphor bronze, the temperature rise when an electrical current is applied is small, making it easier to pass more current.

It was shown that a rapidly solidified Al-Fe alloy could be used as a conductive material with superior sustainability compared to the conventional copper materials. Suitable applications include applications where weight reduction is important, such as in smartphones and wearable devices. The properties of the Al-Fe alloy are adjusted by increasing the extrusion temperature and annealing, and this mechanism was clarified by the present study.

## Data Availability

Not applicable.
